# Impact of CytoSorb and CKRT on hemodynamics in pediatric patients with septic shock: the PedCyto study

**DOI:** 10.3389/fped.2023.1259384

**Published:** 2023-09-15

**Authors:** Gabriella Bottari, Isabella Guzzo, Andrea Cappoli, Raffaella Labbadia, Salvatore Perdichizzi, Carmela Serpe, Jacques Creteur, Corrado Cecchetti, Fabio Silvio Taccone

**Affiliations:** ^1^ Pediatric Emergency Department, Pediatric Intensive Care Unit, Bambino Gesù Children's Hospital, IRCCS, Rome, Italy; ^2^ Department of Pediatrics, Division of Nephrology and Dialysis, Bambino Gesù Children's Hospital, IRCCS, Rome, Italy; ^3^ Department of Intensive Care, Hopital Universitaire de Bruxelles (HUB), Université Libre de Bruxelles (ULB), Brussels, Belgium

**Keywords:** blood purification, children, intensive care, organ dysfunction, vasopressors, septic shock, hemoadsorption, CytoSorb

## Abstract

**Background:**

There is a lack of data to support the use of hemoadsorption in pediatric septic shock. The aim of our study was to assess the effectiveness and safety of CytoSorb therapy in this setting.

**Methods:**

Phase II interventional single arm pilot study in which 17 consecutive children admitted with septic shock who required continuous kidney replacement therapy (CKRT) and weighed ≥10 kg were included. A CytoSorb (CytoSorbents Inc, New Jersey, USA) hemoadsorption cartridge was added to the CKRT every 24 h for a maximum of 96 h. A control group of 13 children with septic shock treated with CKRT but not hemoadsorption at Children's Hospital Bambino Gesù and enrolled in the EuroAKId register was selected as an historical cohort. The primary outcome of the study was a reduction in vasopressor or inotrope dose of >50% from baseline by the end of CytoSorb therapy. Secondary outcomes included hemodynamic and biological changes, changes in severity scores, and 28-day mortality.

**Results:**

There were significant decreases in the Vasoactive Inotropic Score (VIS) and the Pediatric Logistic Organ Dysfunction 2 (PELOD-2) score at 72 and 96 h from the start of the CytoSorb therapy compared to baseline; the reductions were larger in the hemoadsorption group than in the control group (historical cohort). 28-day mortality was lower, although not significantly, in the hemoadsorption group when compared to the control group (5/17 [29%] vs. 8/13 [61%] OR 0.26 [95% CI: 0.05–1.2]; *p* = 0.08).

**Conclusions:**

CytoSorb therapy may have some benefits in pediatric patients with septic shock*.* Future larger randomized trials are needed in this setting.

**Clinical Trial Registration:**

https://clinicaltrials.gov/ct2/show/NCT05658588, identifier (Clinicaltrials.gov NCT05658588).

## Introduction

Septic shock is a life-threatening disease in children and is considered the main cause of death from infection in childhood (1). The majority of children who die from sepsis have refractory shock and/or multiple organ dysfunction syndrome, with most deaths occurring within the first 48–72 h of treatment (1–5). Although improved clinical management, based on early identification, appropriate resuscitation, and adequate antibiotic therapy, has contributed to reducing mortality rates over the last 5 years, up to 50% of children with sepsis, will eventually die from septic shock, depending on underlying risk factors and geographic location (6–9).

Many authors have advocated the need for adjuvant therapies in pediatric septic shock (10), although the evidence supporting this approach is quite limited and the most recent guidelines on the management of pediatric septic shock did not recommend any such strategies, including extracorporeal blood purification (11). The CytoSorb device is a cartridge intended for direct hemoadsorption that has shown a consistently good safe profile in many studies. It is composed of polystyrene divinylbenzene and polyvinylpyrrolidone copolymers and targets molecules in the 5–50 kDa range, which includes the molecular mass of several cytokines. Compelling evidence from the literature shows that the use of CytoSorb in adult patients with septic shock was associated with a reduction in vasopressor requirements, helping to stabilize hemodynamics (12–14) and leading to shock reversal, particularly in patients with refractory shock (15). However, although some authors have also reported an improvement in survival (16–18), the effects of such therapies remain controversial and relatively limited data on survival are available from randomized trials and other observational studies (19–21).

Evidence on the impact of hemoadsorption in pediatric septic shock is limited to case reports and to retrospective case-series (22, 23). The aim of our study was therefore to assess the effectiveness and safety of CytoSorb therapy in pediatric septic shock, compared to an historical cohort.

## Methods

### Study design and patient selection

Phase II interventional single arm pilot study performed in the pediatric intensive care unit (PICU) of the Bambino Gesù Children's Hospital, Rome, Italy. The study protocol was submitted to the local Ethics Committee and approved in July 2019 (N376). Written informed consent was given by the patient's next of kin or guardian. All patients admitted to the PICU because of microbiologically confirmed or suspected septic shock were screened for eligibility from July 2019 and October 2021.

Inclusion criteria included: (1) weight ≥10 kg; (2) septic shock as defined by the International Pediatric Consensus Conference (24) (Supplementary file); (3) need for continuous kidney replacement therapy (CKRT), either for acute kidney injury, defined by the KDIGO criteria (25), and/or fluid overload ≥10%. Exclusion criteria were refusal of consent by parents or concomitant use of other extracorporeal blood purification techniques.

### Data collection and definition

Data were recorded using an electronic case report form (eCRF). Data collection at admission (enrolment) included demographics, comorbidities, source of admission, primary and secondary diagnoses, the Pediatric Logistic Organ Dysfunction 2 score (PELOD-2), and vasoactive inotropic score (VIS) (26, 27), the Pediatric Index of Mortality 3 (PIM-3) score and the Bedside Refractory Septic Shock Score (brSSS) (2, 28) (Supplementary file). Invasive arterial pressure was continuously measured and recorded every 2 h from baseline (onset of therapy) for 108 h. Cardiac index (CI), and systemic vascular resistance index (SVRI) were obtained using a thermodilution technique integrated with pulse contour analysis technology (PiCCO®, Getinge, Göteborg, Sweden) for at least 108 h. The left ventricular ejection fraction (LVEF) was calculated at baseline and every 24 h for 108 h from the onset of the therapy, by an experienced cardiologist using transthoracic echocardiography and the Simpson approach.

C-reactive protein (CRP), Procalcitonin (PCT), lactate and arterio-venous PCO_2_ gap were measured from arterial and venous gas analyses every 8 h from baseline for 108 h. Doses of vasopressors and inotropes were recorded by nurses every 2 h from baseline for 108 h. PELOD-2 score and VIS were assessed at baseline, 24, 48, 72 and 96 h from the onset of therapy.

### Cytosorb therapy

A hemodialysis catheter was inserted into a central vein (internal jugular or femoral depending on the patient's size); CKRT was performed with a standard hemofilter (polyarylethersulphone or ANST69) combined with CytoSorb (CytoSorb®, CytoSorbents Inc, New Jersey, USA) in continuous venovenous hemodiafiltration (CVVHDF) mode, using a pre-filter reinfusion and an effluent dose of 2,000 ml/h × 1.73 m^2^. The CytoSorb cartridge was inserted in the CKRT circuit in series with the hemofilter, changed every 24 h, and continued for a maximum of 96 h. Both the CKRT circuit and Cytosorb were flushed with saline solution and primed with blood. Anticoagulation was managed with regional citrate anticoagulation with a starting citrate dose of 2.5 mmol/L and with an aim of circuit calcium of 0.3–0.4 mmol/L and patient calcium of 1.1–1.25 mmol/L or with unfractioned heparin with a continuous infusion of 10–20 UI/kg/h to achieve a post-filter activated clotting time (ACT) between 160 and 180 s In patients on extracorporeal membrane oxygenation (ECMO), the CKRT access and return were placed post-oxygenator and pre-pump.

### Study outcomes

The primary outcome of the study was the numbers of patients who achieved >50% reduction in vasopressor or inotrope dose from baseline to the end of treatment (maximum 96 h). Patients who had a >50% reduction in the dose of at least one vasoactive drug during the hemoadsorption treatment were considered “responders”. If a patient died before the end of the study period, he/she was considered a “non-responder”.

Secondary outcomes included: (a) doses of vasopressors and inotropes and VIS score at baseline, 24, 48, 72, 96 and 108 h from the onset of therapy; (b) changes in CI, SVRI, systolic pressure (Psys), diastolic pressure (Pdia), and mean pressure (Pmean) from baseline to 24, 48, 72, 96 and 108 h from the onset of therapy; (c) changes in LVEF over time; (d) time-course of CRP, PCT, lactate, and PCO_2_ gap from baseline, 24, 48, 72, 96 and 108 h from the onset of therapy; (e) changes in PELOD-2 score at baseline, and at 24, 48, 72, 96 h from the onset of therapy; (f) 28-day mortality, mortality at PICU and at hospital discharge, and lengths of PICU and hospital stays.

### Historical cohort

**Figure 1:**
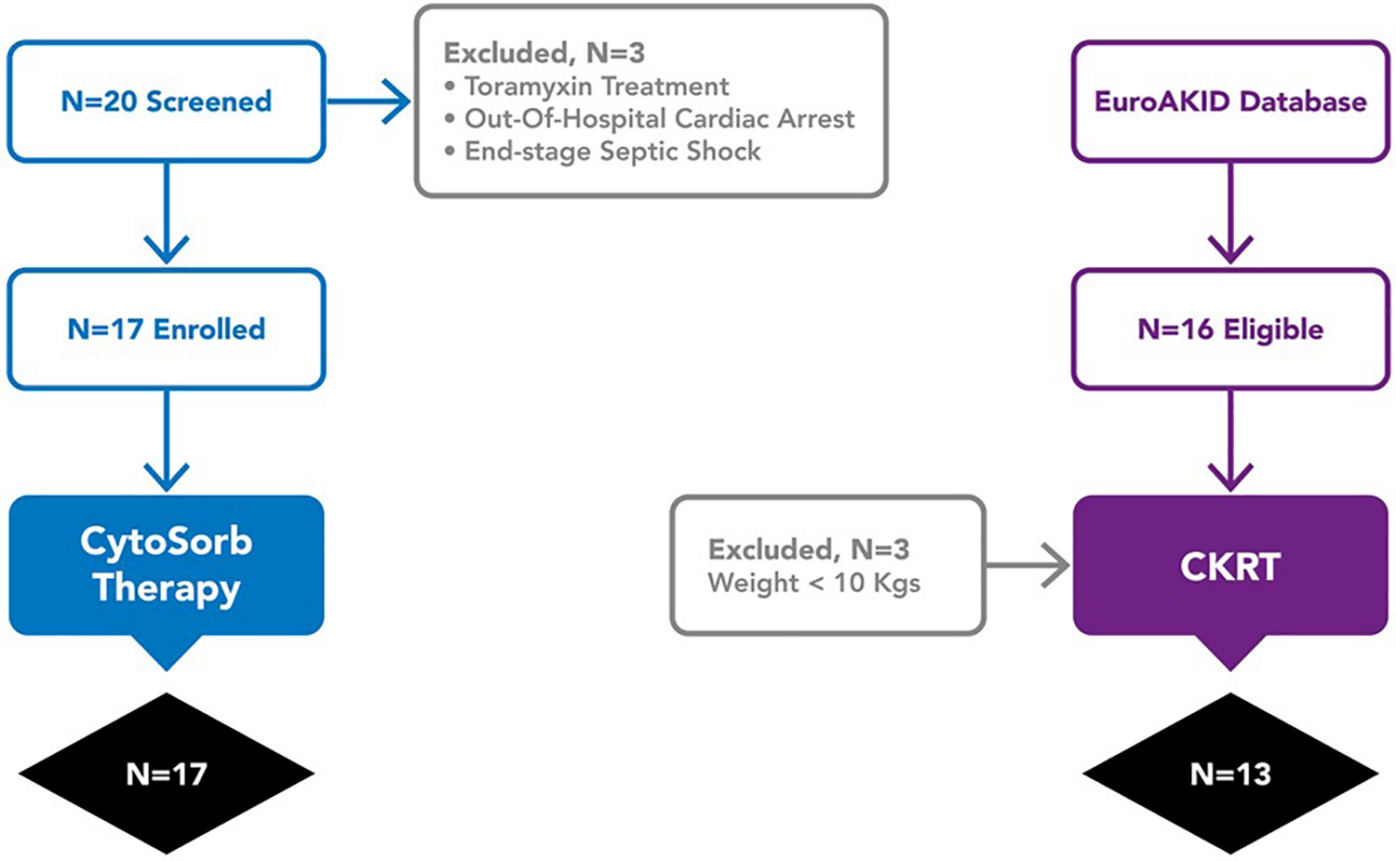
Study flow chart.

An hystorical cohort as control group was created from all cases of pediatric septic shock who received CKRT for acute kidney injury defined by the KDIGO criteria and/or ≥10% fluid overload and who were treated at Children's Hospital Bambino Gesù and enrolled in the EuroAKId register (29) between 2016 and 2018. Sixteen patients met these criteria; three were excluded because of a body weight less than 10 kg (Figure 1). In the two groups, we compared VIS score, the PELOD-2 score at 72 and 96 h from the start of extracorporeal treatment (CKRT alone or CKRT plus hemoadsorption), and mortality at 28 days.

### Statistical analysis

Based on our clinical experience reported in a previous retrospective study regarding hemoadsorption in pediatric septic shock (23), we planned to test the null hypothesis that the true response rate was >50% reduction in vasopressor and/or inotrope dose within 96 h from the start of Cytosorb therapy in 50% of the patients treated, against the alternative hypothesis that the true response rate would be observed in 90% of the patients treated, with a significance level *α* of 5% and a power of 1 − *β* of 80%. Thus according to Simon's optimal design 17 patients had to be enrolled. During the first stage, 2 patients were enrolled with a limit for the first stage rejection of the null hypothesis of 1 responder patient. During the second stage following 15 patients were enrolled with a limit for the second stage of 9 responders patients.

For descriptive data, continuous variables are expressed as mean ± standard deviation (SD) or median and interquartile range (IQR), according to their distribution; categorical variables are expressed as count (%). Mortality at 28 days was evaluated between the two groups with a log-rank test to compare distribution. A *p* value less than 0.05 was considered statistically significant. All statistical analyses were performed using XLSTAT excel advanced statistical software (Version 2022.3, Addinsoft Inc, Paris, France) and NCSS 2021 Statistical Software (NCSS, LLC. Kaysville, Utah, USA).

## Results

### Population characteristics

From 20 eligible patients admitted during the study period, 3 were excluded (one was moribund after cardiac arrest; one was being treated with Polimixin B-cartridge CKRT; one patient had limitations to life-sustaining therapies) and 17 patients with septic shock were therefore enrolled. Median age and weight were 9 (3–13) years and 35 (15–51 kg, respectively). Nine of the 17 patients (52%) had a hemato-oncological disease as a comorbidity, including immune-deficiency and/or a malignancy; two patients had post-surgical septic shock (Table 1). The most frequent primary pathogens were Gram positive bacteria (7/17, 41%) and in 5 (35%) patients a second pathogen was identified. On PICU admission, the median PIM-3 was 98.6 (25.4–99.9)% and the median brSSS was 2 (1–4). All patients were treated with invasive mechanical ventilation; one patient also received venous-arterial ECMO before the onset of septic shock.

**Table 1:** Demographic characteristics of the intervention and control groups.

	CytoSorb (*n* = 17)	Control (*n* = 13)
Age, years	9 [3–13]	6 [3–11]
Weight, kgs	35 [15–51]	20 [13–32]
Male, *n* (%)	8 (47)	11 (85)
brSSS score on admission	2 [1–4]	1 [0–1]
PIM-3 score on admission (%)	98.6 [25.4–99.9]	NA
Cardiac arrest before PICU, *n* (%)	4 (24)	2 (15)
Comorbidities		
Hemato-oncological diseases	9	7
Post-surgical septic shock	2	3
Others	2	3
Sepsis characteristics		
Source of infection		
Primary bacteremia	6	7
Gastrointestinal	3	1
Respiratory	3	1
Abdominal	2	4
Meningo-encephalitis	1	–
Other	3	–
Primary pathogen		
Bacteria (Gram positive)	7	5
Bacteria (Gram negative)	3	5
Virus	4	1
Fungus	3	1
Secondary pathogen		
Bacteria (Gram positive)	1	1
Bacteria (Gram negative)	2	–
Virus	1	4
Fungus	1	1
On the day of assessment		
Vasopressors, *n* (%)	17 (100)	13 (100)
Sedation, *n* (%)	17 (100)	13 (100)
Invasive mechanical ventilation, *n* (%)	17 (100)	13 (100)
PELOD-2 score	9 [7–11]	9 [5–11]
VIS score	47 [36–71]	42 [22–69]

PIM-3, pediatric index of mortality 3; brSSS, bedside refractory septic shock score; PELOD-2, pediatric logistic organ dysfunction 2; VIS, vasoactive inotropic score; NA, not available.

### Characteristics of the hemoadsorption treatment

All treated patients received hemoadsorption within 24 h after the onset of septic shock. The median number of cartridges used per patient was 3 (range: 3–5) and the median duration of hemoadsorption was 72 (range: 63–96) h.

### Primary endpoint

All treated patients received at least 2 vasoactive drugs; a significant reduction (>50%) in the initial dose by the end of the treatment (maximum 96 h) of at least one vasopressor or inotropic agent, characterizing these patients as responders, was achieved in 14 of the 17 patients, specifically for norepinephrine in 14/17 patients, for epinephrine in 13/17 patients and for vasopressin in 2/4 patients.

### Secondary endpoints

**Figure 2:**
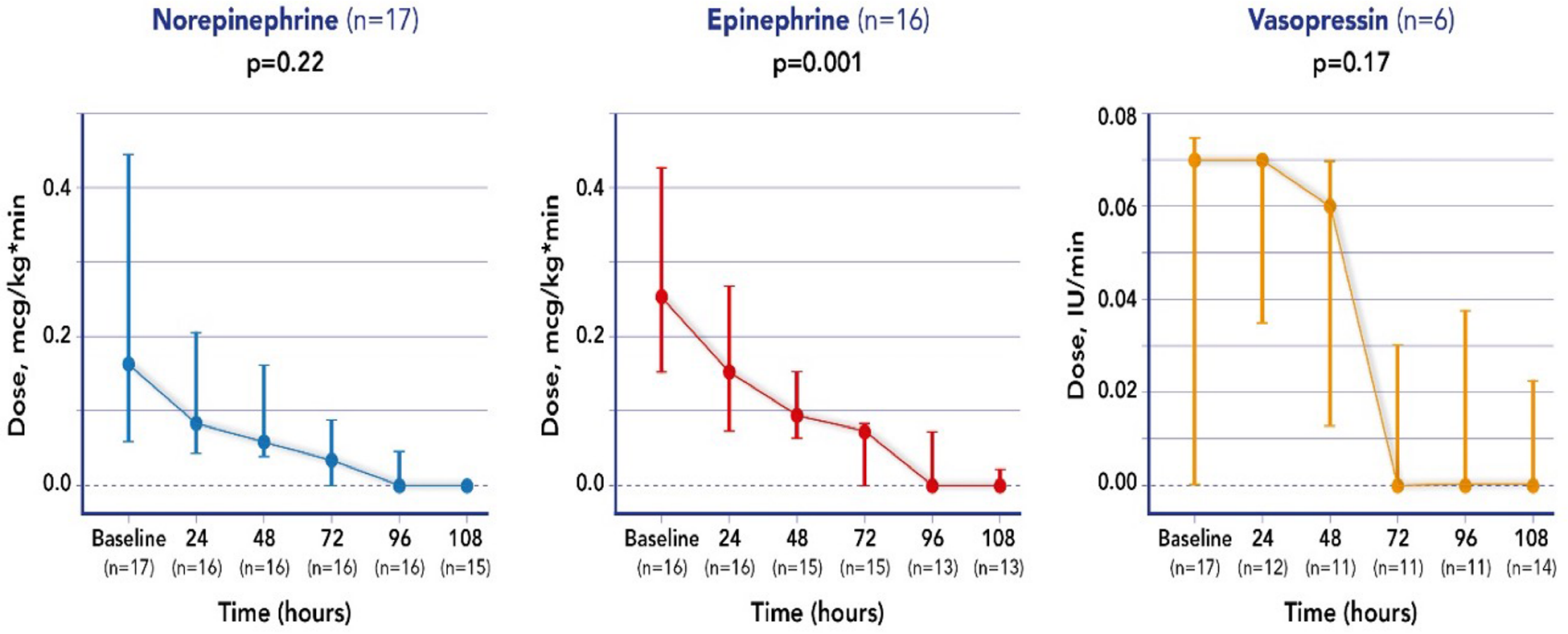
Changes in vasopressor dose over time, from the start of CytoSorb therapy (baseline).

**Figure 3:**
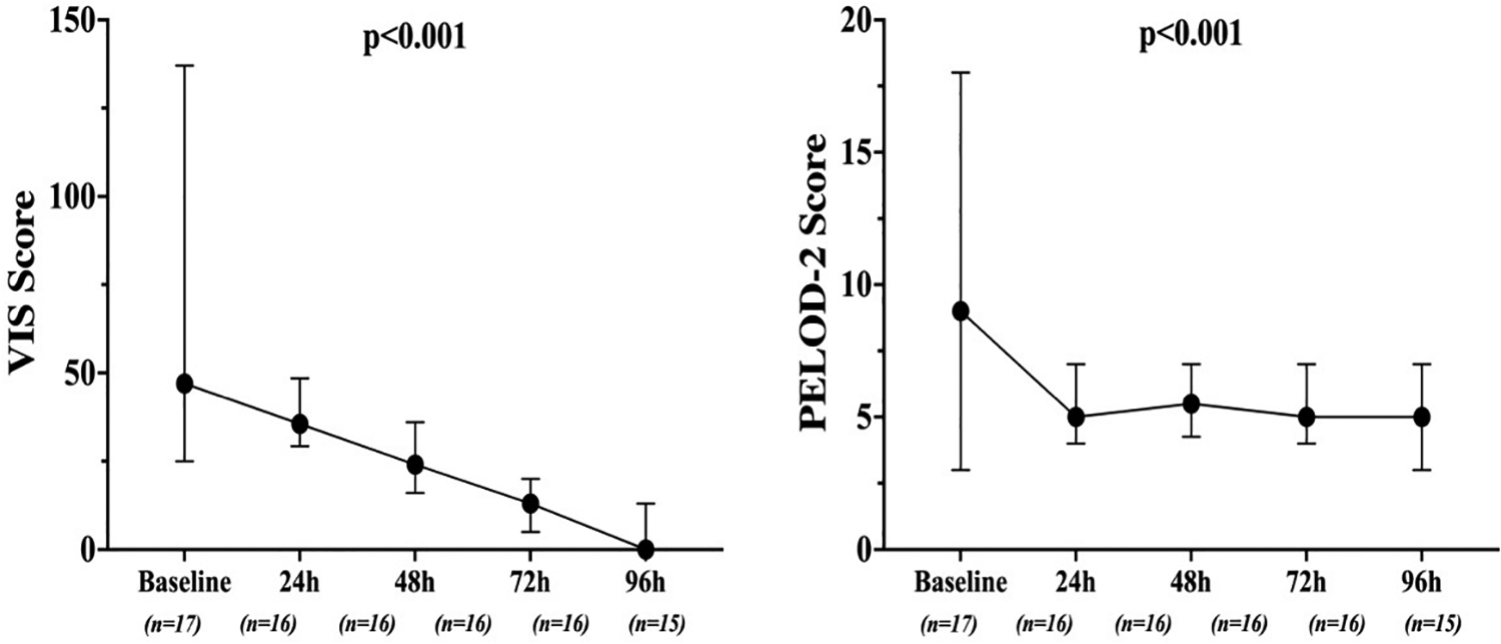
Changes in VIS and PELOD-2 scores over time, from the start of CytoSorb therapy.

Mean doses of epinephrine, but not norepinephrine or vasopressin, were significantly reduced over time, compared to baseline (Figure 2). VIS and PELOD-2 scores also showed a significant reduction over time when compared to baseline values (*p* < 0.001 for both—Figure 3). Table 2 shows the values for the main hemodynamic parameters, perfusion indices and biomarkers of infection over time during hemoadsorption treatment; blood pressure values increased significantly whereas blood lactate levels and CRP concentrations decreased significantly over time compared to baseline values before the initiation of hemoadsorption.

**Table 2:** Time course of main variables.

	Baseline (*n* = 17)	24 h (*n* = 15)	48 h (*n* = 14)	72 h (*n* = 14)	96 h (*n* = 14)	108 h (*n* = 11)	*p* Value
Cardiac index, L/min	3 [2.2–4.4]	3.8 [3.0–4.2]	4.0 [3.0–4.6]	3.8 [3.3–4.5]	3.52 [3.2–4.3]	4.5 [3.6–4.8]	0.36
SVRI, dyn*sec m^2^/cm^2^	1,217 [690–1,599]	1,370 [1,173–2,138]	1,541 [1,205–1,953]	1,378 [1,090–1,608]	1,597 [1,380–1,872]	1,275 [1,106–1,587]	0.65
SBP, mmHg	79 [71–102]	111 [98–120]	108 [102–112]	110 [99–118]	110 [105–119]	115 [106–122]	<0.001
DAP, mmHg	41 [35–57 ]	59 [50–71]	55 [50–66]	62 [53–72]	60 [53–70]	61 [57–71]	0.02
MAP, mmHg	56 [47–70]	78 [65–86]	77 [71–85]	78 [70–88]	80 [70–92]	83 [75–89]	0.001
Lactate, mmol/L	5.8 [2–8]	1.5 [1–2.3]	1.5 [0.9–2.1]	1.3 [1.1–1.8]	1.3 [1–1.5]	1 [0.8–1.1]	0.006
PCO_2_ gap, mmHg	5 [4–7]	6 [4–7]	6 [4–7]	4 [3–7]	6 [5–7]	5 [3–6]	0.48
PCT, ng/ml	30 [21–102]	34 [11–59]	11[6–24]	11 [6–19]	7 [0.7–28]	1.11 [0–5]	0.09
CRP, mg/dl	26 [20–30]	23 [18–28]	25 [19–29]	19 [18–24]	13 [7–18]	11.35 [9–16]	0.03

Data are presented as median and range.

T, time; SVRI, systemic vascular resistance index; PCT, procalcitonin; CRP, C-reactive protein; SBP, systolic blood pressure; DAP, diastolic blood pressure; MAP, mean arterial pressure; Baseline, onset of Cytosorb therapy.

The LVEF did not change significantly over time overall, but improved in those patients with initial myocardial dysfunction (*n* = 9; Supplementary Figures S1, S4) (30). Mortality at 28 days was 29% (5/17), at PICU discharge was 35% (6/17) and at hospital discharge 47% (8/17). Median lengths of PICU and hospital stay were 16 (8–41) and 30 (18–73) days, respectively.

### Comparison with the control group

**Figure 4:**
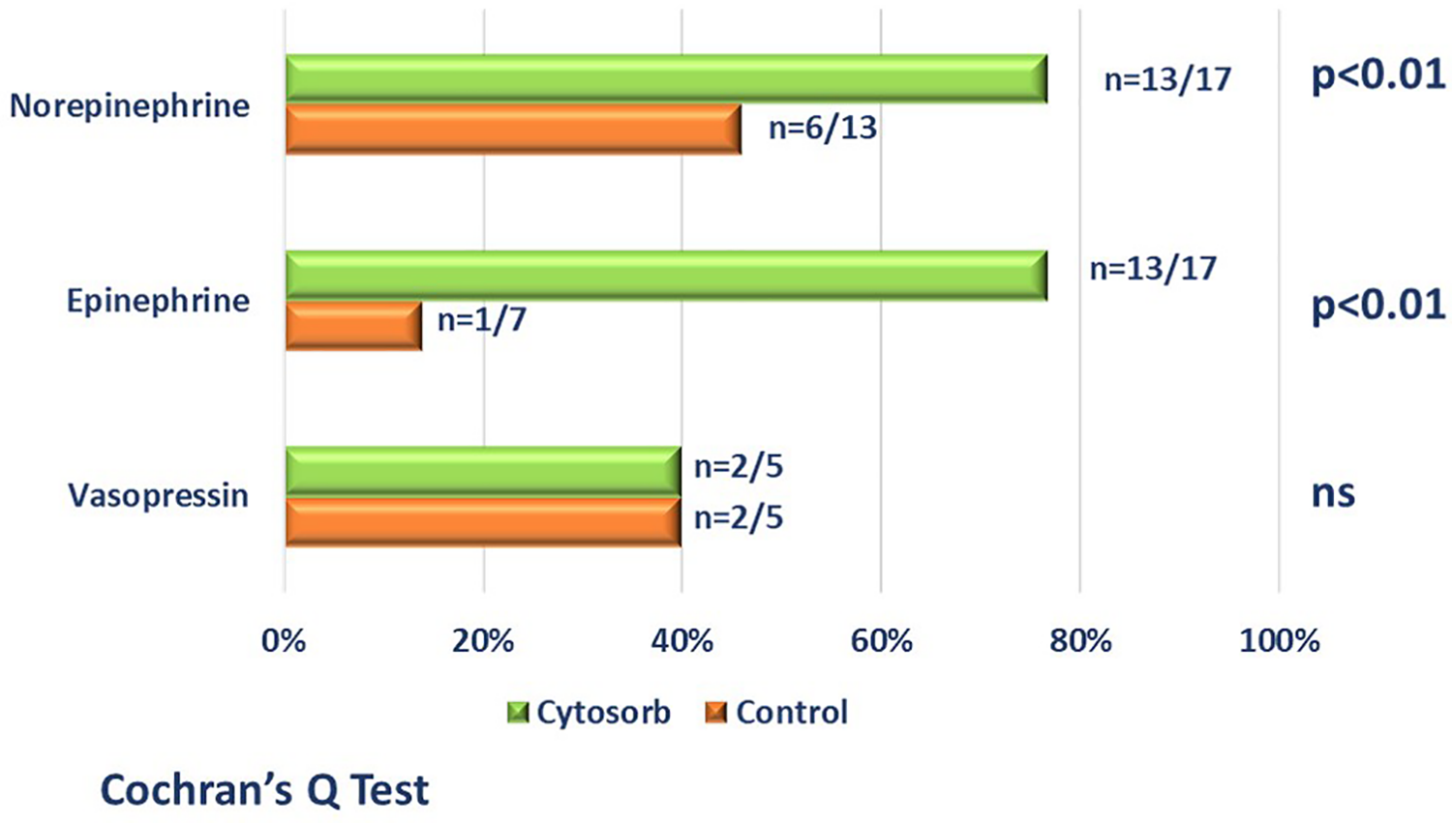
Comparison (percentage and absolute number) of responders (patients who achieved a reduction >50% of at least one vasoactive drugs by 96 h from the start of blood purification) in the hemoadsorption group [cytosorb plus CKRT-green bars] and in control group [CKRT alone-orange bars]. Patient who died before the end of the study period, were considered non-responders.

**Figure 5:**
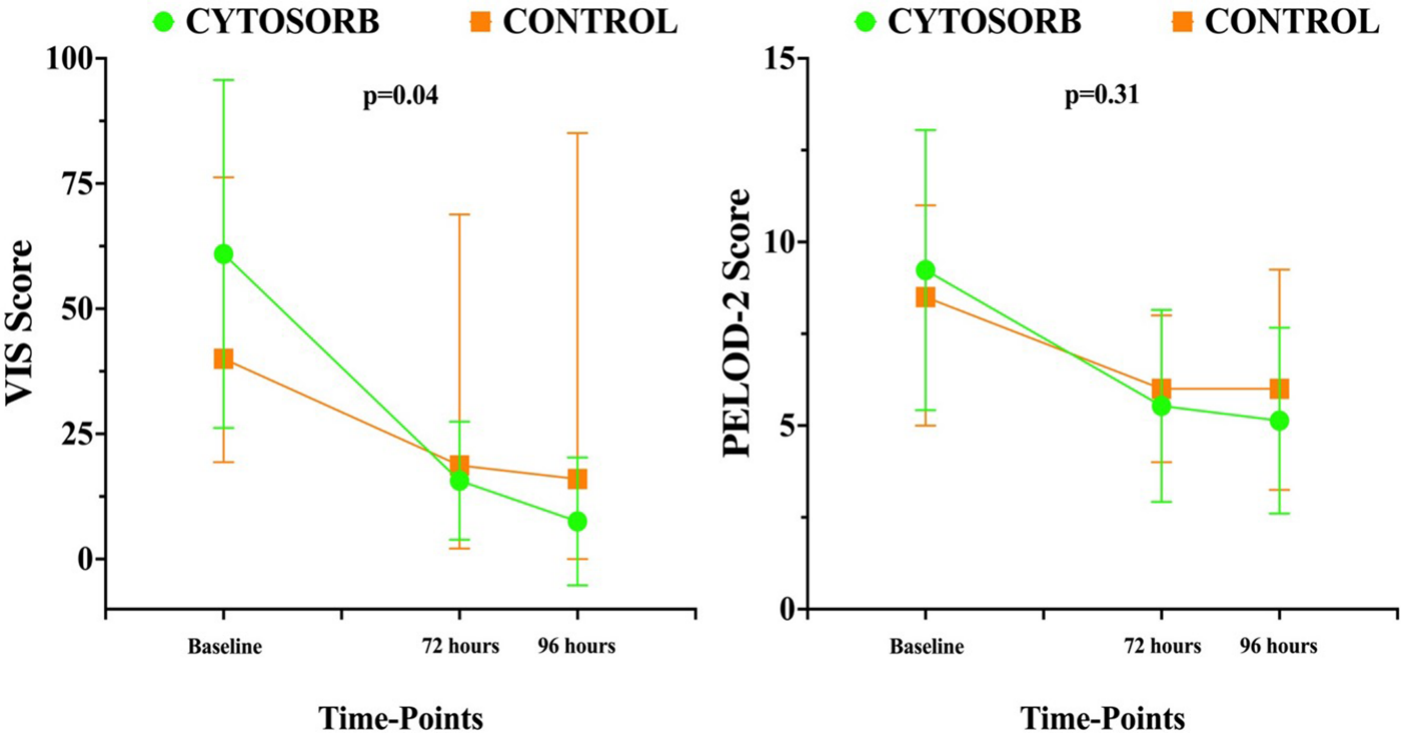
Time course of pediatric logistic organ dysfunction (PELOD) 2 score and vasoactive inotropic score (VIS) over time in the two groups. Data are shown as median and range.

Table 1 reports the demographic characteristics, comorbidities and sources of infection in the treated and control groups (historical cohort). Figure 4 shows a comparison of responders (percentage and absolute number) in the treatment group (CKRT and hemoadsorption) and in the control group (CKRT alone). There was a significantly larger reduction in the VIS in the hemoadsorption group than in the control group, over time, but not in the PELOD-2 score (Figure 5). Table 3 reports characteristics of patients (age, gender and comorbidities) in relation to the primary outcome (responders vs. non responders) and to the 28-day mortality in the two groups. 28-day mortality was lower, although not significantly, in the hemoadsorption group when compared to the control group in all patients (5/17 [29%] vs. 8/13 [61%] OR 0.26 [95% CI: 0.05–1.2]; *p* = 0.08).

**Table 3:** Reports characteristics of patients (age, gender and comorbidities) in responders vs. non responders patients (defined as reduction of at least one vasoactive drugs by 96 h from the start of CKRT and hemoadsorption [Cytosorb group] or CKRT alone [control group], if a patient died before the end of the study period, he/she was considered a “non-responder”).

Cytosorb	Non responder	Responder	Survived	No survived
17 patients	3	14	12	5
Age, years	2 [2–14]	10 [2–17]	12 [2–17]	3[2–12]
Male, *n* (%)	3 (3)	6 (14)	5 (12)	3 (5)
Comorbidities				
Hemato-oncological disease	2 (3)	6 (14)	4 (12)	3 (5)
Chronic disease	1 (3)	1 (14)	1 (12)	1 (5)
Post-surgical septic shock		2 (14)	2 (12)	
Control	Non responder	Responder	Survived	No survived
13 patients	6	7	5	8
Age, years	8 [2–16]	3 [2–16]	4 [2–16]	7.5 [2–16]
Male, *n* (%)	3 (6)	6 (7)	4 (5)	5 (8)
Comorbidities				
Hemato-oncological diseases	5 (6)	2 (7)		7 (8)
Chronic disease	1 (6)	2 (7)	2 (5)	2 (8)
Post-surgical septic shock		1 (7)		

The same characteristics are reported also in relation to survival at 28 days in the two groups (survived vs. no survived patients).

### Adverse events

No hypotensive events were observed after the initiation of therapy. Two cardiac arrests leading to deaths occurred within 24 h from the initiation of therapy; after review by the safety monitoring board, they were not considered as device-related, but secondary to the disease severity. At the end of the treatment, hemoglobin and platelets variations from baseline values were 3% (from −7% to 14%) and 26% (from −7% to 71%), respectively (Supplementary Figures S2, S7).

## Discussion

Use of CytoSorb therapy in pediatric septic shock patients was associated with a significant decrease in vasopressor requirements over time. Blood pressure, blood lactate levels and inflammatory parameters also improved over time. Compared to a untreated control group, use of such therapy was associated with better hemodynamic control and numerically lower mortality rate. No serious adverse events related to the device were reported.

Other authors have already studied blood purification therapies in pediatric septic shock; Kawai et al. (31) used therapeutic plasma exchange (TPE) in children with multiple organ dysfunction syndrome (MODS) due to sepsis and requiring ECMO, showing an improved VIS and organ failure index after a median of three TPE cycles. Polymyxin-B hemoadsorption with PMX-05 cartridges was applied in 15 children with septic shock; the authors reported a trend towards an improvement in hemodynamics after two sessions of therapy, although inotropic agent requirements did not change over time (32). More recently, Saetang et al. described the use of PMX-20R (a modified polymyxin-B cartridge circuit for pediatric use) in 6 children and reported a significant reduction in PELOD-2 and VIS scores after two sessions of therapy (33). Clinical experiences are also reported on application of the Selective Cytopheretic Device (SCD) in critically ill children with AKI and multiorgan dysfunction showing promising results in terms of safety and efficacy (34).

Following previous clinical studies on efficacy of blood purification in pediaric septic shock (23, 35) we have established a reduction >50% in vasoactive drugs from the start of extracorporeal treatment, as threshold of adequate response to the treatment.

In our study, we analyzed a larger cohort of patients with severely impaired systemic hemodynamics, as suggested by the very high VIS score at the onset of hemoadsorption; after initiation of blood purification there was a significant improvement in the hemodynamic status and a reduction in vasopressor requirements, suggesting that hemoadsorption might have a role as rescue therapy in these patients. Despite the high initial disease severity, none of our patients required rescue venous-arterial ECMO, because of rapid improvement after blood purification treatment, which is in contrast with recent evidence reporting a quite frequent use of such therapy in severe pediatric septic shock (36). In our population only one patient in the treatment group received venous-arterial ECMO, but the VA ECMO was started before the onset of septic shock for the primary disease, instead in the historical cohort one patient received rescue V-A ECMO for a clinical picture of refractory septic shock.

As in studies evaluating CytoSorb use in the adult population, early initiation of treatment (13, 37) and a large number of sessions (38) appeared to be the optimal setting for such therapy. In our cohort, therapy was initiated within 24 h from the onset of septic shock; this is not always feasible in randomized trials, as the need for written consent often delays the initiation of therapy beyond 48–72 h from the onset of shock. Furthermore, the high adsorption surface area (>40,000 m^2^) of CytoSorb, when compared to the body surface and the blood volume of paediatric patients, might have a larger impact than in adult patients to modulate the inflammatory response and therefore improve cardiovascular function.

The improvement in organ dysfunction was less significant than the reduction in vasopressor doses, which is in contrast with previous studies conducted in adult populations, showing a significant improvement in the sequential organ failure assessment (SOFA) score after the initiation of hemoadsorption (39). The lack of a significant improvement in PELOD-2 score could be related both to the high incidence of comorbidities in our population and to the small cohort enrolled. Therefore further assessment of organ function is required in larger populations. Regarding the impact of hemoadsorption on myocardial dysfunction, it deserves attention that significant improvement in LVEF% was observed in those patients with a severe to moderate dysfunction suggesting a beneficial role of blood purification on myocardial stunning induced by cytokine storm which could recognize the same beneficial mechanism of immunomodulation for the reduction of VIS score in our population.

We cannot draw any conclusion on the differences between CytoSorb and other blood purification techniques; however, as patients in the control group received CKRT, we can consider that blood purification is more effective at stabilizing hemodynamics than is conventional CKRT, although further investigations using different membranes (e.g., higher cut-off) and CKRT doses might be required. To avoid center-bias, we selected from the EuroAKId database only patients treated in the Children Hospital Bambino Gesù and however our control group is an historical one, there were no major changes of management of children with septic shock and CKRT which could affect the outcomes, except for an higher implementation of regional anticoagulation with citrate. With all the limitations of a very small cohort, we observed a more rapid reduction in vasopressor requirements and a lower mortality rate in the hemoadsorption group.

It is also important to underline that the observed 28-day mortality in the hemoadsorption group was much lower than was expected using the brSSS score and PIM-3 (i.e., >50%) (2); moreover, our cohort included a high proportion of patients with hemato-oncological disease (9 of 17), which is independently associated with an increased probability of death and might impact long-term outcome, even after sepsis has been controlled (40). Finally, no serious safety concerns were observed during therapy. These results highlight the need for large phase-III studies to further evaluate safety and feasibility and better quantify mortality when hemoadsorption is rapidly implemented in pediatric septic shock patients.

Our study has some limitations. First, it was a single-arm, single-center interventional study, using an historical cohort as a control group, which might result in some methodological biases and overestimate the effects of therapy. Second, as therapeutic strategies were not entirely standardized, we cannot exclude that other confounders (e.g., early and adequate antibiotic therapy, fluid resuscitation, source control) might also influence our findings.

## Conclusions

To the best of our knowledge, this is the first interventional study describing the use of hemoadsorption therapy with CytoSorb cartridges in children with septic shock. The use of hemoadsorption was associated with a significant reduction in vasopressor requirements over time, compared to baseline values and to a control group. Potential benefits on survival were also observed. No major safety issue was reported. Multicenter studies are warranted to confirm these initial promising findings.

## Data Availability

The raw data supporting the conclusions of this article will be made available by the authors, without undue reservation.

## Supplementary material

Supplementary Figure S4 Changes in left ventricular ejection fraction (LVEF) over time in all patients and in those with initial systolic myocardial dysfunction (30).

Supplementary Figure S7 Cohort platelet count (×10^3^/mcl) and hemoglobin dosage (gr/dl) during the time of CKRT plus Cytosorb hemoadsorption treatment. Platelets counts and hemoglobin dosage were obtained prior the onset of hemoadsorption at baseline (T0), after 24 h (T1) and at the end of hemoadsorption treatment (T2). The horizontal lines represent the median count, boxes the IQR.
