# Supplementary material for: Impact of CytoSorb and CKRT on hemodynamics in pediatric patients with septic shock: the PedCyto study
Source: Front Pediatr. 2023 Sep 15;11:1259384. doi: 10.3389/fped.2023.1259384 (PMC10540853; doi:10.3389/fped.2023.1259384)
Supplement: Supplementary file 1 [file Datasheet1.pdf]

Supplementary file.

1. Definitions of systemic inflammatory response syndrome (SIRS), infection, sepsis, severe sepsis, and septic shock according to International pediatric sepsis consensus conference (24)

Systemic inflammation response syndrome (SIRS)

The presence of at least two of the following four criteria, **one of which must be abnormal temperature or leukocyte count:**

- Core temperature of >38.5°C or < 36°C.

- Tachycardia, defined as a mean heart rate □2 SD above normal for age in the absence of external stimulus, chronic drugs, or painful stimuli; or

otherwise unexplained persistent elevation over a 0.5- to 4-hr time period **OR for children <1 yr old: bradycardia, defined as a mean heart rate <10th percentile for age in the absence of external vagal stimulus, beta-blocker drugs, or congenital heart disease; or otherwise unexplained persistent depression over a 0.5-hr time period.**

- Mean respiratory rate > 2 SD above normal for age or mechanical ventilation for an acute process not related to underlying neuromuscular disease or the receipt of general anesthesia.

- Leukocyte count elevated or depressed for age (not secondary to chemotherapy-induced leukopenia) or >10% immature neutrophils. *Infection*

A suspected or proven (by positive culture, tissue stain, or polymerase chain reaction test) infection caused by any pathogen OR a clinical syndrome associated with a high probability of infection. Evidence of infection includes positive findings on clinical exam, imaging, or laboratory tests (e.g., white blood cells in a normally sterile body fluid, perforated viscus, chest radiograph consistent with pneumonia, petechial or purpuric rash, or purpura fulminans)

Sepsis

SIRS in the presence of or as a result of suspected or proven infection.

Severe sepsis

Sepsis plus one of the following: cardiovascular organ dysfunction OR acute respiratory distress syndrome OR two or more other organ

Septic shock

Sepsis **and cardiovascular organ dysfunction**

2. **Bedside refractory septic shock score (2)**

– Bedside SSS (bSSS) based on 5 points with coefficient ranked and rounded to have a user-friendly score:
– VIS > 200 mcg/kg min = 1 point
– Arterial lactate >8 mmol/L or its increase of 1 mmol/L after 6 h of care = 1 point
– Myocardial dysfunction as defined above = 3 points. Myocardial dysfunction, defined as the occurrence of a resuscitation-responsive cardiac arrest in PICU or car- diac ultrasound findings with left ventricle ejection fraction (LVEF) <25 % or a cardiac index <2.2 L/min m^2.

3. **VIS score (26).**

The vaso-inotrope dependency was assessed with the use of the vaso-inotrope score [VIS = (epinephrine + norepi- nephrine in mcg/kg min) × 100 + (dobutamine + dopa- mine in mcg/kg min) + (vasopressin in mcg/ kg min) × 10,000 + (milrinone in mcg/kg min) × 20]

4. Pelod-2 score (27)

Table I - Pediatric Logistic Organ Dysfunction Score - (PELOD-2)[6]

**Organ dysfunctions and variables**	**Points by severity level**						
	**0**	**1**	**2**	**3**	**4**	**5**	**6**
Neurologic							
• Glasgow coma score	≥11	5-10			3-4		
• Pupillary reaction	Both reactive					Both fixed	
Cardiovascular							
• Lactatemia (mmol/L)	<5.0	5.0-10.9			≥11.0		
• Mean arterial pressure (mmHg) (months)							
0-<1	≥ 46		31-45	17-30			≤ 16
1-11	≥ 55		39-54	25-28			≤ 24
12-23	≥ 60		44-59	31-43			≤ 30
24-59	≥ 62		46-61	32-44			≤ 31
60-143	≥ 65		49-64	36-48			≤ 35
≥144	≥ 67		52-66	38-51			≤ 37
Renal							
• Creatinine (μmol/L) (months)							
0-<1	≥ 69		≥ 70				
1-11	≥ 22		≥ 23				
12-23	≥ 34		≥ 35				
24-59	≥ 50		≥ 51				
60-143	≥ 58		≥ 59				
≥144	≥ 92		≥ 93				
Respiratory							
• PaO_2 (mmHg)/FiO_2	≥ 61		≤ 60				
• $PacO_2$ (mmHg)	≥ 58	59-94		≥ 95			
• Invasive ventilation	No			Yes			
Hematologic							
• WBC Count ($x10^9$/L)	>2		≤ 2				
• Platelet ($x10^9$/L)	≥142	77-141	≤ 76				

Relationship between number of organ dysfunctions, PELOD-2 score, and mortality rate

Number of organ dysfunctions	PELOD-2 Score Mean (SD)	Mortality rate (%)
0	0 (0.0)	0.4
1	2.3 (0.8)	0.3
2	4.9 (1.3)	1.2
3	7.5 (2.0)	7.1
4	11.5 (4.4)	30.5
5	16.8 (5.2)	59.0

SD = Standard deviation
