# Supplementary figures and images for: Impact of CytoSorb and CKRT on hemodynamics in pediatric patients with septic shock: the PedCyto study
Source: Front Pediatr. 2023 Sep 15;11:1259384. doi: 10.3389/fped.2023.1259384 (PMC10540853; doi:10.3389/fped.2023.1259384)

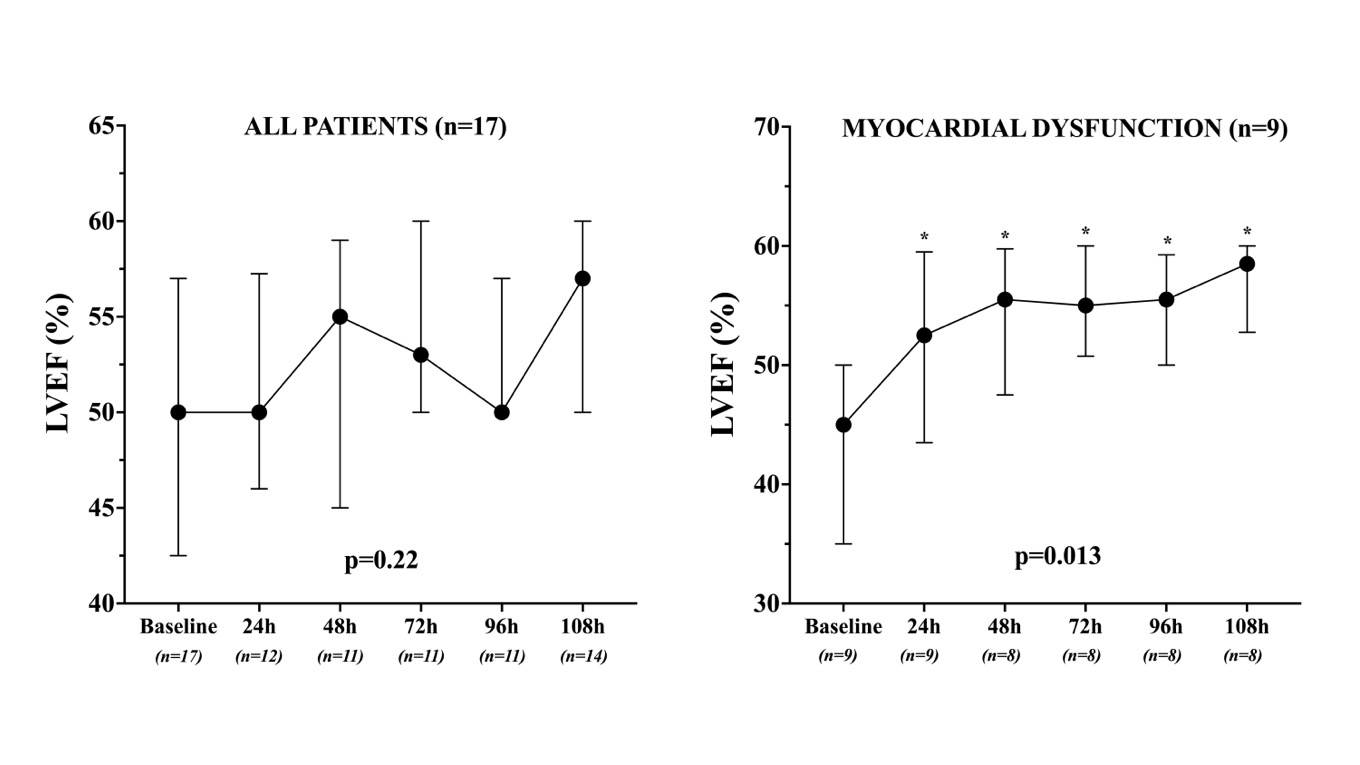

Supplement: Supplementary Figure S4 — Changes in left ventricular ejection fraction (LVEF) over time in all patients and in those with initial systolic myocardial dysfunction (30). [file Image1.jpeg]

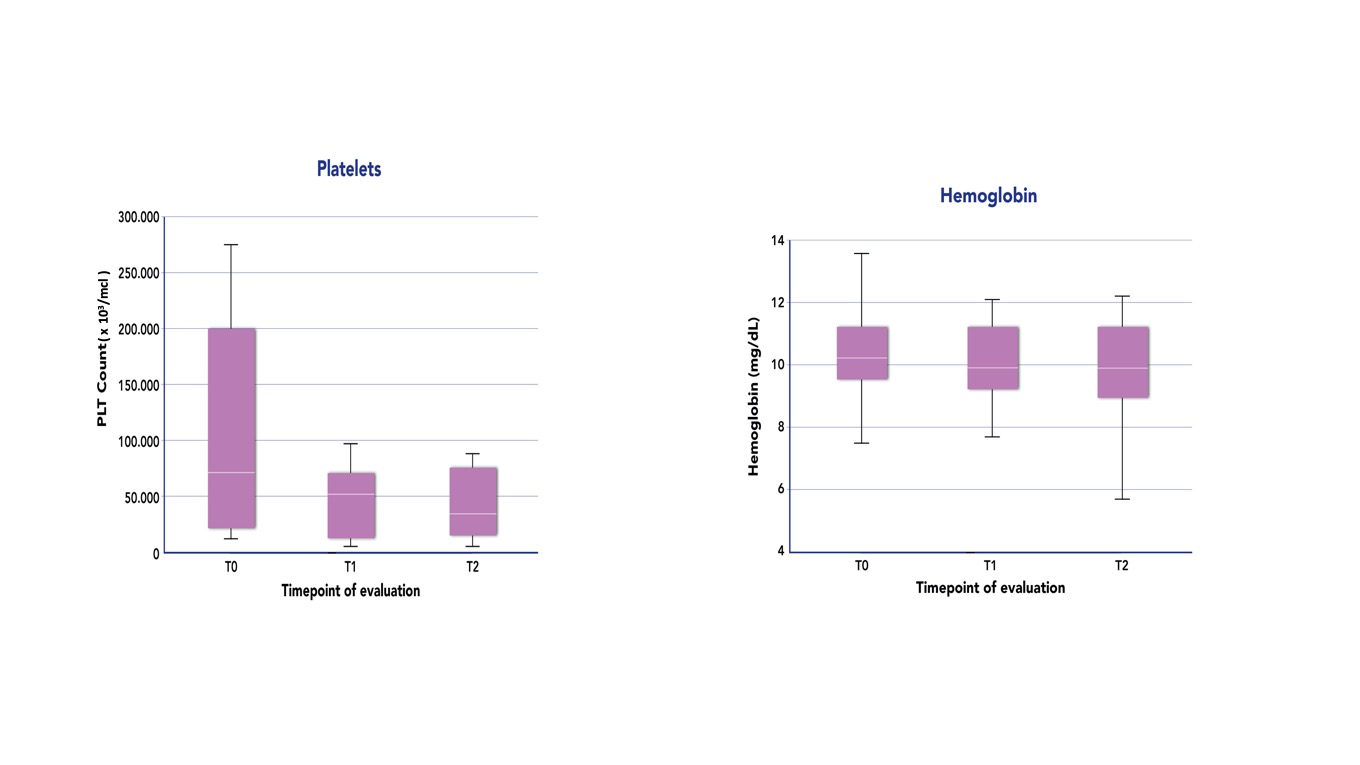

Supplement: Supplementary Figure S7 — Cohort platelet count (×103/mcl) and hemoglobin dosage (gr/dl) during the time of CKRT plus Cytosorb hemoadsorption treatment. Platelets counts and hemoglobin dosage were obtained prior the onset of hemoadsorption at baseline (T0), after 24 h (T1) and at the end of hemoadsorption treatment (T2). The horizontal lines represent the median count, boxes the IQR. [file Image2.jpeg]
